# The causal relationship between rheumatoid arthritis and interstitial lung disease in East Asian population: A two-sample Mendelian randomization study

**DOI:** 10.1097/MD.0000000000039903

**Published:** 2024-10-04

**Authors:** Xiang Sun, Peipei Huang, Lingshan Gao, Weixing Zhong, Lixia Yuan

**Affiliations:** a School of Traditional Chinese Medicine, Southern Medical University, Guangzhou, China.

**Keywords:** causal effect, interstitial lung disease, Mendelian randomization, rheumatoid arthritis

## Abstract

To investigate the causal relationship between rheumatoid arthritis (RA) and interstitial lung disease (ILD) in the East Asian population, we utilized Mendelian randomization (MR). Publicly available summarized data from genome-wide association studies on RA (4199 cases and 208,254 controls), and the data on ILD (1046 cases and 176,974 controls) were obtained from BioBank Japan. Eligible single nucleotide polymorphisms from East Asian populations were obtained from genome-wide association studies as instrumental variables, and 11 RA-related single nucleotide polymorphisms (*P* < 5 × 10^−8^) were selected as instrumental variables. MR analysis was performed using inverse variance weighted, MR-Egger regression, weighted median, and MR-PRESSO with RA as the exposure data and ILD as the outcome data. Reliability was evaluated using Cochran *Q* test, MR-Egger intercept, leave-one-out analysis, and funnel plot. Inverse variance weighted results showed an odds ratio (95% confidence interval) of 1.29 (1.18–1.41), *P* = 3.99 × 10^−8^, indicating a positive association between RA and ILD. The reliability evaluation could adopt the fixed-effect model, and the absolute value of the MR-Egger regression intercept was 0.021, *P* > 0.05, and *P* value of Global Test in MR-PRESSO was 0.573. The test results of the leave-one-out showed that the results are robust, and the funnel plot indicated that the instrumental variables were not affected by potential factors. In conclusion, this study demonstrates that RA is a risk factor for ILD in the East Asian population.

## 1. Introduction

Rheumatoid arthritis (RA) is a chronic autoimmune inflammatory disease characterized by synovial inflammation, hyperplasia, production of autoantibodies, and destruction of articular cartilage.^[1]^ According to the Global Burden of Diseases, Injuries, and Risk Factors Study, about 383 million people worldwide will die from RA in 2020, and the mortality rate varies by region. It seriously endangers people’s physical and mental health and brings a heavy burden to the social economy.^[2]^ According to relevant reports, RA patients have an increased risk of cardiovascular, gastrointestinal, respiratory and other diseases, and lung disease is the second cause of RA death.^[3]^ Interstitial lung disease (ILD) encompasses a group of diffuse parenchymal lung diseases with high morbidity and mortality, influenced by various factors including environment, genetics, inflammation, and fibrosis.^[4]^ RA is the most common extraarticular manifestation of ILD, with the incidence ranging from 7.7% to 67%.^[5]^ RA may be associated with the occurrence of ILD.^[6]^ However, the causal relationship between RA and ILD is limited by confounding factors and reverse causality.

ILD is the most common lung problem in RA patients. Epidemiological studies have found that about 14.7% of RA patients develop ILD, which can cause various lung issues.^[7]^ Prospective observations have shown that around 32.5% of RA patients without respiratory symptoms previously have lung abnormalities, with 18.8% having poor lung function and 7.5% being diagnosed with ILD.^[8]^ However, some studies suggest that ILD might appear before RA, with early RA patients exhibiting autoantibodies in sputum antibodies before joint symptoms.^[9,10]^ Previous studies may have different conclusions due to confounding factors, and using Mendelian randomization (MR) can reduce biases and provide more accurate conclusions.

Usual interstitial pneumonia is a characteristic pattern of lung injury in idiopathic pulmonary fibrosis (IPF), occurring in approximately 40% of individuals with RA-associated interstitial lung disease (RA-ILD). Recent studies have utilized two-sample bidirectional MR to investigate the potential causal relationship between RA and usual interstitial pneumonia. IPF has demonstrated a significant causal effect on seropositive RA, with the development of IPF increasing the risk of seropositive RA. In the opposite direction, MR analysis revealed a significant protective effect of seropositive RA on IPF; however, this effect was not significant when sensitivity analyses were conducted. The findings of this MR study differ from our conclusions, which is that RA directly increases the risk of ILD in East Asian population. This discrepancy may be attributed to the fact that the study population was primarily European, and genetic susceptibility varies among different populations.^[11]^

MR is a statistical method that utilizes genetic variants strongly associated with exposure factors, known as instrumental variables, to infer causal effects between exposures and outcomes. By leveraging the genome-wide association study (GWAS) data, MR reduces the bias from confounding factors and reverse causation and provides a temporal order of causation.^[12]^ In this study, a two-sample MR approach was used to explore the potential causal relationship between RA and ILD in the East Asian population. The findings aim to guide clinical practice, improve prognosis identification, and enhance patient survival.

## 2. Methods

### 2.1. Study design

**Figure 1.**
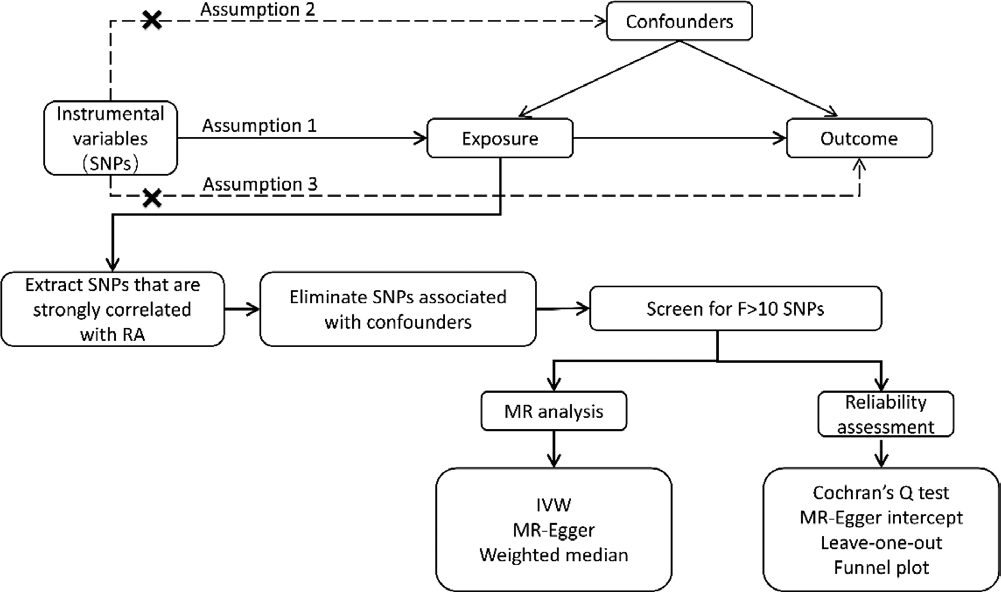
Study the design flow chart.

This study used RA as the exposure variable and ILD as the outcome variable. Instrumental variables were obtained from the East Asian population GWAS genetic summary dataset, ensuring the independence of exposure and outcome variables. MR analysis requires meeting 3 main hypotheses:^[13]^ (1) correlation assumption: there is a significant correlation between single nucleotide polymorphisms (SNPs) and RA; (2) independence assumption: SNPs and confounders are not correlated with each other; (3) exclusion assumption: SNPs can only exert an effect on ILD through RA (Fig. 1).

### 2.2. Data sources

We sourced SNPs linked to RA and ILD from the IEU Open GWAS database, which houses publicly accessible aggregated GWAS data. Specifically, exposure data on RA (dataset: bbj-a-151; cases: 4199, controls: 208,254, PMID: 32514122) and outcome data on ILD (dataset: ebi-a-GCST90018643; cases: 1046, controls: 176,974, PMID: 34594039) were extracted from BioBank Japan, where ethical approval and informed consent were secured (Table 1). The Japan Biobank, our data source, is the largest in East Asia, encompassing information on 42 prevalent diseases from approximately 200,000 individuals aged 20 to 89 years. The research adhered to protocols endorsed by the respective institutional ethics committees, with informed consent from participants, and was additionally approved by the ethics committees of both the Riken Research Institute Center for General Medical Science and the Institute of Medical Sciences, University of Tokyo.

**Table 1:** Data sources for this study of exposures and outcomes in GWAS.

Trait	Data source	ID	Population	Sample size	Cases	Controls	SNP	Year	PMID
Rheumatoid arthritis	https://gwas.mrcieu.ac.uk/	bbj-a-151	East Asian	212,453	4199	208,254	8885,805	2019	32514122
Interstitial lung disease	https://gwas.mrcieu.ac.uk/	ebi-a-GCST90018643	East Asian	178,020	1046	176,974	12,454,608	2021	34594039

To mitigate potential biases, including winner’s curse and weak instrument bias due to sample overlap from the BBJ, we performed a sensitivity analysis using only 3 SNPs that met a stringent GWAS significance threshold (*P* < 1 × 10^−13^), known to reduce these biases compared to the conventional threshold (*P* < 5 × 10^−8^).^[14–16]^

### 2.3. Selection of SNPs

SNPs for MR analysis must exhibit significant GWAS with RA (*P* < 5 × 10^−8^) to meet the association criteria. Linkage disequilibrium (*r*^2^ = 0.001, kb = 10,000) was tested to ensure independence by excluding SNPs with linkage imbalance. Then, in order to satisfy the hypothesis and avoid SNPs being associated with confounders or ILDs, the PhenoScanner database was used to find the relevant phenotypes of SNPs, and SNPs associated with confounders and ILDs were manually eliminated. Additionally, SNPs (A/T or C/G) that were not palindromic with effect allele frequencies between 40% and 70% were selected. Finally, according to the assumptions of MR analysis, in order to ensure a strong correlation between IVs and exposure, the intensity of the selected SNPs was assessed, and the F value of individual SNPs was calculated to rule out possible weak IV bias between IVs and exposure factors. If F > 10, there is little chance of bias for weak IVs. The formula is as follows: $F=\frac{R^{2}\left(n-k-1\right)}{k\left(1-R^{2}\right)}$</mathgraphic>, *R*^*2*^ is the proportion of IV variation explained by SNPs, k is the number of IVs included in the study, and n is the sample size.

### 2.4. MR analysis

Inverse variance weighted (IVW), MR-Egger regression, and weighted median methods were used for MR analysis. IVW calculated the Wald ratio for each instrumental variable, combining the results through inverse variance weighted analysis.^[17]^ The slope of the IVW method could explain the causal effect of RA on ILD, indicating that the exposure and outcome were statistically significant when *P* < 0.05.

### 2.5. Reliability assessment

Cochran *Q* test assessed heterogeneity in the effects of RA-related SNPs on ILD outcomes, favoring the fixed-effect model when *P* > 0.05. MR-Egger regression tested for potential pleiotropy and assessed result robustness. A close-to-zero absolute value of the Egger regression intercept with *P* > 0.05 indicated no horizontal pleiotropy.^[18]^ The MR-PRESSO further detected whether there is horizontal pleiotropy and abnormal SNPs with pleiotropic effects and then provided estimated values after removing outliers. There is no horizontal pleiotropy when the *P* value of Global Test is >0.05.^[19]^ In addition, leave-one-out sensitivity analysis tested the influence of individual instrumental variables on the IVW estimate. A consistent combined effect of other IVs with the main effect suggested no undue effect on the MR analysis. Results were expressed as odds ratio (OR) and 95% confidence interval (95% CI), with a *P* < 0 .05 considered statistically significant. The “Two-Sample-MR” package in R software (version 4.3.1) was utilized for all statistical analyses, with α = 0.05 as the significance level.

## 3. Results

### 3.1. Instrumental variables

Following the screening criteria, 12 strongly correlated SNPs without linkage imbalance were identified from the RA exposure data. The PhenoScanner database confirmed the absence of phenotypes related to confounders or ILDs in these SNPs. Individual SNP F-values ranged from 32.39 to 694.46, indicating no weak instrumental variables. After eliminating 1 SNP (rs3093017) during effect allele alignment, 11 SNPs were included as instrumental variables to assess the causal association between RA and ILD (Table 2).

**Table 2:** SNPs eventually included in this study.

SNPs ID	CHR	EA	OA	EAF	β	SE	*P*	F value
rs117530403	6	G	T	0.13	1.03	0.04	4.76 × 10^−153^	694.46
rs12612769	2	C	A	0.30	0.15	0.02	1.06 × 10^−9^	37.20
rs1557549	6	G	A	0.13	–0.49	0.04	5.64 × 10^−35^	152.22
rs1634734	6	A	G	0.45	–0.18	0.02	6.27 × 10^−15^	60.81
rs2082260	6	T	G	0.54	0.13	0.02	7.48 × 10^−9^	33.41
rs3757387	7	C	T	0.11	0.21	0.04	1.58 × 10^−18^	34.26
rs56139217	10	C	T	0.11	0.21	0.04	4.83 × 10^−9^	32.39
rs77117142	6	T	C	0.06	–0.34	0.05	1.26 × 10^−8^	48.14
rs79658451	6	C	G	0.12	0.21	0.04	3.97 × 10^−12^	34.53
rs80202727	6	T	C	0.24	0.19	0.03	4.19 × 10^−9^	49.07
rs9275610	6	C	T	0.33	–0.36	0.02	2.47 × 10^−12^	217.83

CHR = chromosome, EA = effect allele, OA = other allele, SE = standard error.

### 3.2. MR analysis

**Figure 2.**
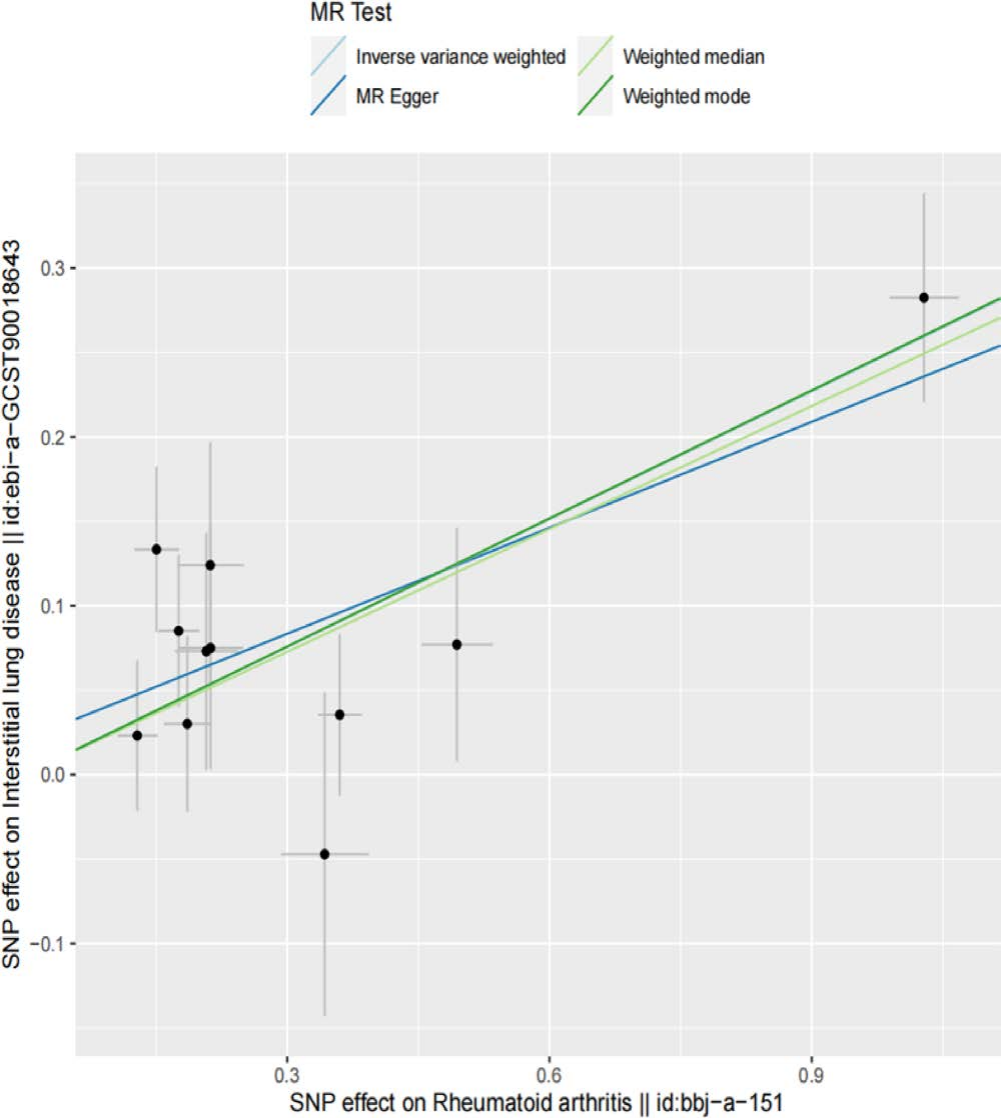
Scatter plot of rheumatoid arthritis and interstitial lung disease.

**Figure 3.**
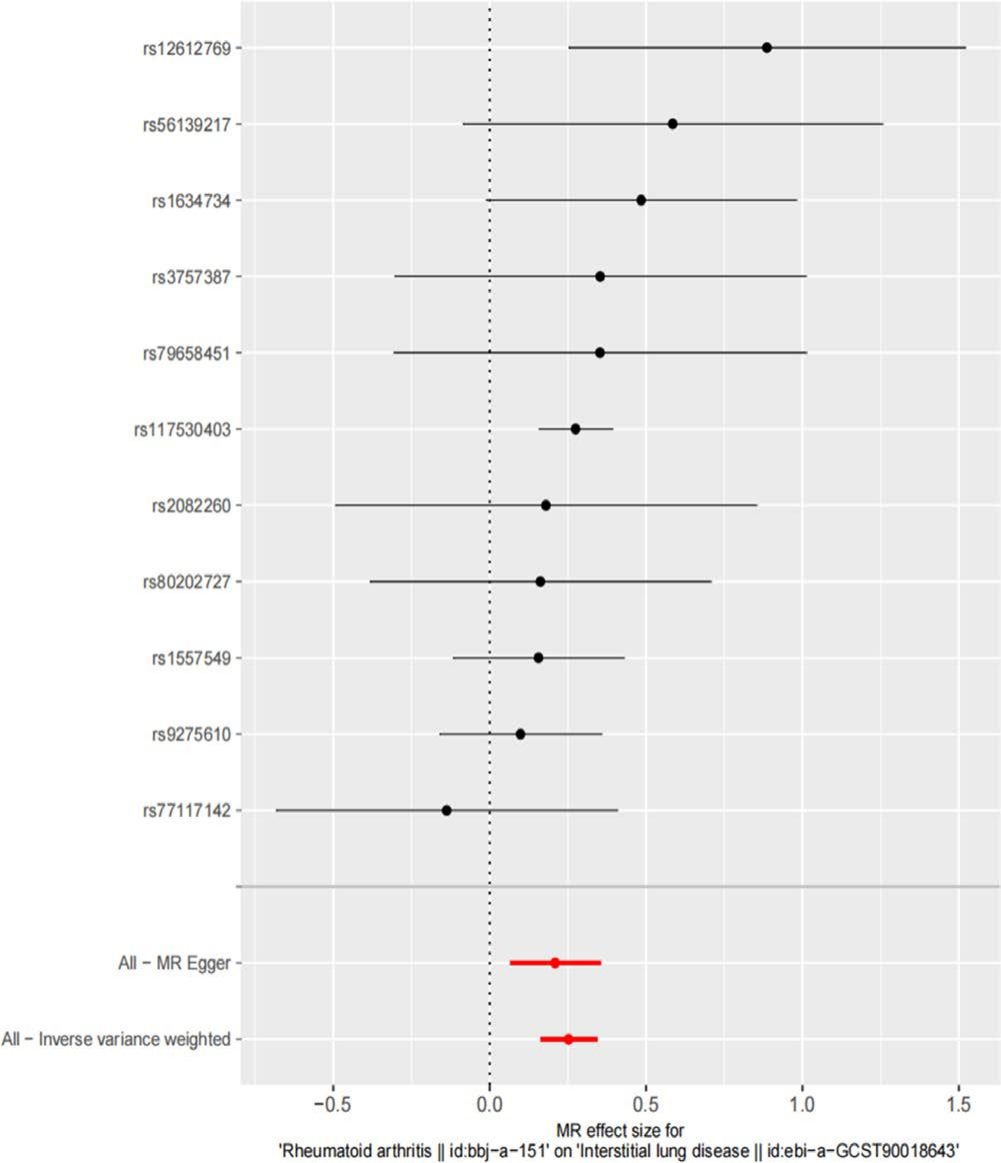
Forest plot of SNP effect size.

In this MR Analysis, fixed-effect model of IVW was adopted, and the results were as follows, the IVW method was OR = 1.29, 95% Cl = 1.18 to 1.41, *P* = 3.99 × 10^−8^, the MR-Egger analysis was OR = 1.23, 95% Cl = 1.07 to 1.42, *P* = 0.02, and the weighted median analysis was OR = 1.27, 95% Cl = 1.14 to 1.42, *P* = 1.73 × 10^−5^, all of which indicated that there may be positive causal effects between RA and ILD in genetic prediction and the *P* value reached statistical significance (Table 3, Figs. 2 and 3).

**Table 3:** Results of two-sample MR.

MR analysis	SNP number	β	SE	OR	95% Cl	*P*
IVW	11	0.25	0.05	1.29	1.18–1.41	3.99 × 10^−8^
MR-Egger	11	0.21	0.07	1.23	1.07–1.42	0.02
WM	11	0.24	0.06	1.27	1.14–1.42	1.05 × 10^−5^

To further investigate the causal relationship, we conducted a sensitivity analysis including only 3 SNPs with a stringent GWAS threshold (*P* < 1 × 10^−13^). The positive causal relationship between RA and ILD persisted, as indicated by the fixed-effects IVW method (OR: 1.39, 95% CI: 1.16–1.68, *P* = 0.0005), weighted median (OR: 1.38, 95% CI: 1.16–1.68, *P* = 1.11 × 10^−5^), and weighted mode (OR: 1.38, 95% CI: 1.19–1.60, *P* = 0.05). However, the MR-Egger analysis yielded different results (OR: 1.38, 95% CI: 0.77–2.47, *P* = 0.48).

### 3.3. Reliability assessment

**Figure 4.**
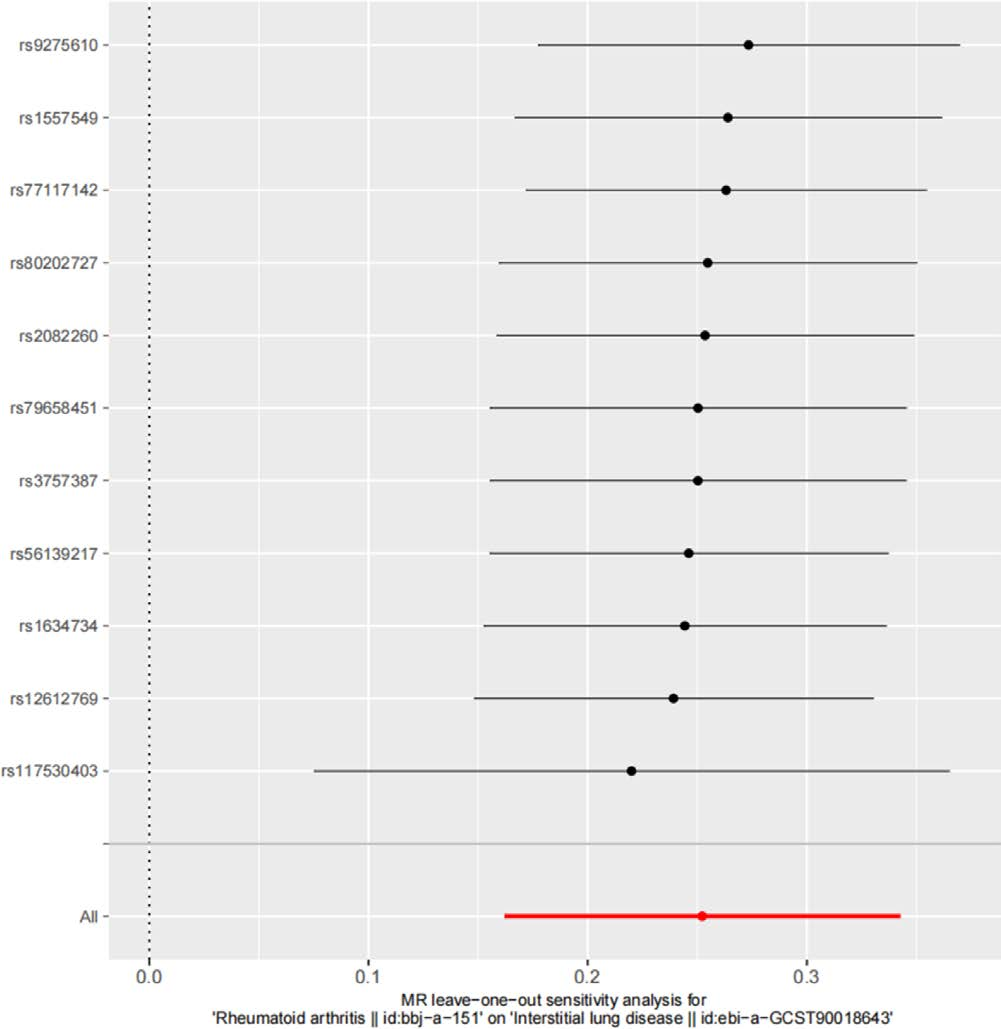
Results of “leave-one-out” sensitivity analysis.

**Figure 5.**
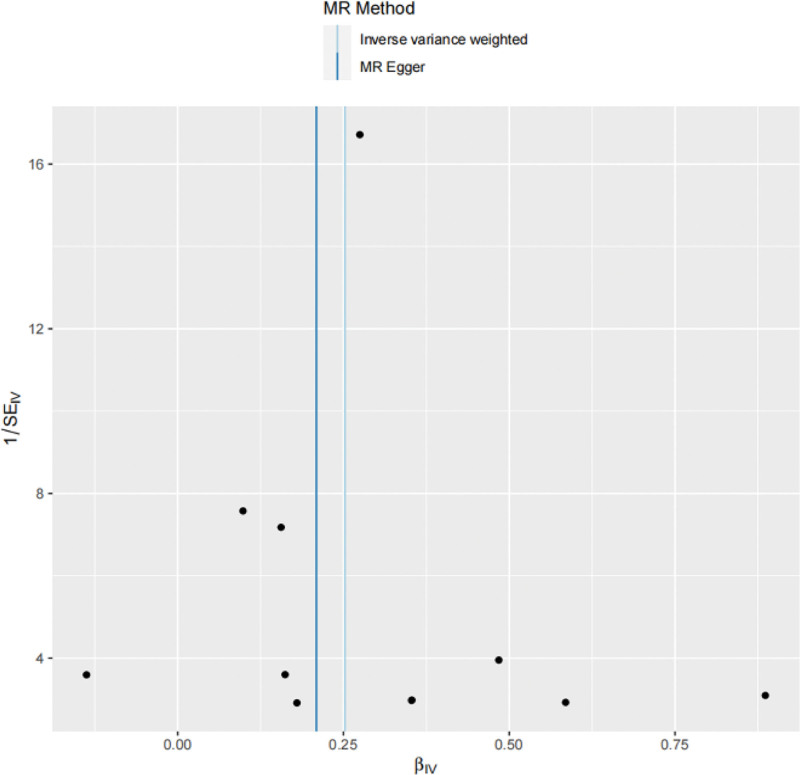
Funnel plot of causal associations.

Cochran *Q* test revealed no significant heterogeneity between SNPs in RA (*P* > 0.05). Therefore, this study used a fixed-effect model to evaluate the potential causal relationship between RA and ILD. MR-Egger regression results showed an absolute value of the regression intercept of 0.021 with *P* > 0.05. The *P* value of Global Test in MR-PRESSO analysis was 0.573, and no outliers are detected so the Distortion Test was not required. These results suggested that no horizontal pleiotropy affected causality. Finally, leave-one-out sensitivity analysis and funnel plot both confirmed the robustness of the results, indicating no bias from potential factors (Figs. 4 and 5).

## 4. Discussion

This study employed Mendelian randomization analysis using the East Asian population GWAS database to investigate the causal effect of genetic predisposition to RA on ILD. The results of the IVW method show that (OR = 1.29, 95% Cl: 1.18–1.41, *P* = 3.99 × 10^−8^), indicating a direct causal relationship between RA and ILD in the East Asian population. RA patients showed a considerable increase in ILD risk compared to the general population, with a 1.29 times higher risk according to the IVW method. The consistent findings from the 3 estimation methods (IVW, weighted median, and MR-Egger regression) support this conclusion. Systematic evaluation and meta-analysis have reported a combined prevalence of 11% (95% CI: 7–15%) for RA combined with ILD.^[20]^ In summary, RA increases the risk of ILD.

ILD is recognized as the most common extraarticular manifestation of RA. Epidemiological studies have reported an ILD incidence of 14.7% among RA patients, with various pulmonary manifestations such as rheumatoid nodules and pleural effusion.^[7]^ Prospective observations have revealed lung abnormalities in 32.5% of RA patients without respiratory symptoms, with 18.8% exhibiting significantly impaired lung function and 7.5% being diagnosed with ILD, indicating the risk of ILD in early RA patients.^[8]^ Retrospective studies have shown a high prevalence of ILD in RA patients, with the imaging results correlating with gender, RA serological indicators, and other factors.^[21]^ Additionally, ILD incidence among RA patients is approximately 5%, and the mortality rate for RA-ILD patients is nearly 4 times higher than that of the general population.^[22]^ However, studies have also suggested that ILD may precede RA, with early RA patients exhibiting autoantibodies in sputum even before arthritis symptoms.^[9,10]^ Inconsistent conclusions from previous observational studies may stem from confounding factors and reverse causation issues, highlighting the advantage of MR in minimizing these biases and providing more reliable causal inferences.^[23]^

Through this MR study, we establish a causal association between RA and ILD in the East Asian population, highlighting the increased risk of ILD in RA patients. Previous studies have identified several mechanisms linking RA and ILD. The inflammatory mediator IL-17A significantly influences RA onset and may mediate pulmonary fibrosis by regulating inflammatory response, fibroblast activity, and NF-κB and STAT3 pathways.^[24]^ RA may impact the development of lung tissue protein citrulline and the production of anti-citrulline protein antibodies through peptidyl arginine deiminase.^[25]^ Immune complex deposition in lung tissue, inducing an inflammatory response, plays a role in the development and progression of RA-ILD.^[26–28]^ RF-IgA complexes may contribute to lung injury, inflammation, and fibrosis.^[29]^ Additionally, reduced peripheral blood memory B cells and lymph accumulation around bronchi may represent important mechanisms in the pathogenesis of RA-ILD.^[30,31]^ The team of Lai NL found that the reduction of peripheral blood CD4 + T cell and the increase of CD56 + NK cell may constitute an important mechanism of RA-ILD.^[32]^ Ethnic genetic differences are also influential, such as the MUC5B promoter variant (rs35705950) as a significant risk factor for RA-ILD.^[33,34]^

The strengths of this study include a low-heterogeneity East Asian population, ensuring generalizability within the racial context. By utilizing MR, this study reduces biases caused by confounding factors and reverse causality, providing more robust causal inferences. A large sample size of 20,000 enhances the credibility of the study, and open access GWAS data minimizes ethical concerns, saves time and costs, and respects ethical considerations. Robustness and independence assessments, such as heterogeneity tests, leave-one-out sensitivity analysis, and funnel plot analysis, confirm the reliability of the results, supporting the assumptions of independence and exclusivity.

However, limitations remain. The generalizability of the results to other ethnic groups requires further investigation beyond the East Asian population. While the statistical effect values of MR are indicative, combined analysis with observational research and exploration of molecular mechanisms is necessary to confirm causal relationships and biological mechanisms. Furthermore, potential nonlinear or hierarchical effects varying by gender, age, personal habits, and disease severity should be explored given the observed heterogeneity.

Studies have shown that ILD can develop into diffuse pulmonary fibrosis and honeycomb lungs, damaging the pulmonary ventilation function of patients and eventually leading to dyspnea or even respiratory failure.^[35]^ It is obvious that ILD has seriously affected the quality of life and survival time in RA patients. Our study suggests that clinicians should closely monitor respiratory symptoms in RA patients, conduct relevant lung imaging examinations, and enhance screening for ILD-related indicators. Proactive treatment and close follow-up are essential for RA patients with significant respiratory symptoms to manage ILD incidence. This research also offers insights into a new diagnostic and treatment strategy for ILD by studying the combined use of antirheumatic and anti-inflammatory drugs to control ILD occurrence and progression.

## Author contributions

**Conceptualization:** Peipei Huang, Xiang Sun, Lingshan Gao, Weixing Zhong.

**Data curation:** Peipei Huang, Xiang Sun, Lingshan Gao, Weixing Zhong.

**Formal analysis:** Peipei Huang, Xiang Sun, Lingshan Gao, Weixing Zhong.

**Funding acquisition:** Peipei Huang, Xiang Sun, Lingshan Gao, Weixing Zhong.

**Investigation:** Peipei Huang, Xiang Sun, Lingshan Gao, Weixing Zhong.

**Methodology:** Peipei Huang, Xiang Sun, Lingshan Gao, Weixing Zhong.

**Project administration:** Peipei Huang, Xiang Sun, Lingshan Gao, Weixing Zhong.

**Resources:** Peipei Huang, Xiang Sun, Lingshan Gao, Weixing Zhong.

**Software:** Peipei Huang, Xiang Sun, Lingshan Gao, Weixing Zhong.

**Supervision:** Peipei Huang, Xiang Sun, Lingshan Gao, Weixing Zhong, Li Xia Yuan.

**Validation:** Peipei Huang, Xiang Sun, Lingshan Gao, Weixing Zhong, Li Xia Yuan.

**Visualization:** Peipei Huang, Xiang Sun, Lingshan Gao, Weixing Zhong, Li Xia Yuan.

**Writing – original draft:** Peipei Huang, Xiang Sun, Lingshan Gao, Weixing Zhong, Li Xia Yuan.

**Writing – review & editing:** Peipei Huang, Xiang Sun, Lingshan Gao, Weixing Zhong, Li Xia Yuan.

## References

[1] Safiri S Kolahi AA Cross M. Prevalence, deaths, and disability-adjusted life years due to musculoskeletal disorders for 195 countries and territories 1990-2017. Arthritis Rheumatol. 2021;73:702–14. 33150702 10.1002/art.41571

[2] GBD 2021 Rheumatoid Arthritis Collaborators. Global, regional, and national burden of rheumatoid arthritis, 1990-2020, and projections to 2050: a systematic analysis of the Global Burden of Disease Study 2021. Lancet Rheumatol. 2023;5:e594–610. 37795020 10.1016/S2665-9913(23)00211-4 PMC10546867

[3] Figus FA Piga M Azzolin I McConnell R Iagnocco A. Rheumatoid arthritis: extra-articular manifestations and comorbidities. Autoimmun Rev. 2021;20:102776. 33609792 10.1016/j.autrev.2021.102776

[4] Wijsenbeek M Suzuki A Maher TM. Interstitial lung diseases. Lancet. 2022;400:769–86. 35964592 10.1016/S0140-6736(22)01052-2

[5] Dai Y Wang W Yu Y Hu S. Rheumatoid arthritis–associated interstitial lung disease: an overview of epidemiology, pathogenesis and management. Clin Rheumatol. 2021;40:1211–20. 32794076 10.1007/s10067-020-05320-z

[6] Kadura S Raghu G. Rheumatoid arthritis-interstitial lung disease: manifestations and current concepts in pathogenesis and management. Eur Respir Rev. 2021;30:210011. 34168062 10.1183/16000617.0011-2021 PMC9489133

[7] Geng Y Xie X Wang Y. The standardized diagnosis and treatment of rheumatoid arthritis. Chin J Int Med. 2022;61:51–9. 10.3760/cma.j.cn112138-20210616-00426 34979770

[8] Robles-Pérez A Luburich P Bolivar S. A prospective study of lung disease in a cohort of early rheumatoid arthritis patients. Sci Rep. 2020;10:15640. 32973236 10.1038/s41598-020-72768-z PMC7515904

[9] Fischer A Solomon JJ du Bois RM. Lung disease with anti-CCP antibodies but not rheumatoid arthritis or connective tissue disease. Respir Med. 2012;106:1040–7. 22503074 10.1016/j.rmed.2012.03.006 PMC3753791

[10] Willis VC Demoruelle MK Derber LA. Sputum autoantibodies in patients with established rheumatoid arthritis and subjects at risk of future clinically apparent disease. Arthritis Rheum. 2013;65:2545–54. 23817979 10.1002/art.38066 PMC4066465

[11] Leavy OC Kawano-Dourado L Stewart ID. Rheumatoid arthritis and idiopathic pulmonary fibrosis: a bidirectional Mendelian randomisation study. Thorax. 2024;79:538–44. 38649271 10.1136/thorax-2023-220856 PMC11137470

[12] Birney E. Mendelian randomization. Cold Spring Harb Perspect Med. 2022;12:a041302. 34872952 10.1101/cshperspect.a041302 PMC9121891

[13] Grover S Del Greco MF Stein CM. Mendelian randomization. Methods Mol Biol. 2017;1666:581–628. 28980266 10.1007/978-1-4939-7274-6_29

[14] Jiang T Gill D Butterworth AS Burgess S. An empirical investigation into the impact of winner’s curse on estimates from Mendelian randomization. Int J Epidemiol. 2023;52:1209–19. 36573802 10.1093/ije/dyac233 PMC10396423

[15] Burgess S Davies NM Thompson SG. Bias due to participant overlap in two-sample Mendelian randomization. Genet Epidemiol. 2016;40:597–608. 27625185 10.1002/gepi.21998 PMC5082560

[16] Deng Y Wong MCS. Association between rheumatoid arthritis and osteoporosis in Japanese populations: a Mendelian randomization study. Arthritis Rheumatol. 2023;75:1334–43. 37039764 10.1002/art.42502

[17] Bowden J Holmes MV. Meta-analysis and Mendelian randomization: a review. Res Synth Methods. 2019;10:486–96. 30861319 10.1002/jrsm.1346 PMC6973275

[18] Burgess S Thompson SG. Interpreting findings from Mendelian randomization using the MR-Egger method. Eur J Epidemiol. 2017;32:377–89. 28527048 10.1007/s10654-017-0255-x PMC5506233

[19] Verbanck M Chen CY Neale B Do R. Detection of widespread horizontal pleiotropy in causal relationships inferred from Mendelian randomization between complex traits and diseases. Nat Genet. 2018;50:693–8. 29686387 10.1038/s41588-018-0099-7 PMC6083837

[20] Joy GM Arbiv OA Wong CK. Prevalence, imaging patterns and risk factors of interstitial lung disease in connective tissue disease: a systematic review and meta-analysis. Eur Respir Rev. 2023;32:220210. 36889782 10.1183/16000617.0210-2022 PMC10032591

[21] Dinache G Popescu CC Mogoșan C Enache L Agache M Codreanu C. Lung damage in rheumatoid arthritis-a retrospective study. Int J Mol Sci . 2022;24:28. 36613472 10.3390/ijms24010028 PMC9820047

[22] Farquhar HJ Beckert L Edwards AL. Rheumatoid interstitial lung disease in Canterbury, Aotearoa New Zealand - a retrospective cohort study. Semin Arthritis Rheum. 2024;64:152359. 38157761 10.1016/j.semarthrit.2023.152359

[23] Skrivankova VW Richmond RC Woolf BAR. Strengthening the reporting of observational studies in epidemiology using mendelian randomisation (STROBE-MR): explanation and elaboration. BMJ. 2021;375:n2233. 34702754 10.1136/bmj.n2233 PMC8546498

[24] Zhang J Wang D Wang L. Profibrotic effect of IL-17A and elevated IL-17RA in idiopathic pulmonary fibrosis and rheumatoid arthritis-associated lung disease support a direct role for IL-17A/IL-17RA in human fibrotic interstitial lung disease. Am J Physiol Lung Cell Mol Physiol. 2019;316:L487–97. 30604628 10.1152/ajplung.00301.2018

[25] Paulin F Doyle TJ Fletcher EA Ascherman DP Rosas IO. Rheumatoid arthritis-associated interstitial lung disease and idiopathic pulmonary fibrosis: shared mechanistic and phenotypic traits suggest overlapping disease mechanisms. Rev Invest Clin. 2015;67:280–6. 26696331 PMC4690466

[26] McInnes IB Schett G. The pathogenesis of rheumatoid arthritis. N Engl J Med. 2011;365:2205–19. 22150039 10.1056/NEJMra1004965

[27] Derksen VFAM Huizinga TWJ van der Woude D. The role of autoantibodies in the pathophysiology of rheumatoid arthritis. Semin Immunopathol. 2017;39:437–46. 28451788 10.1007/s00281-017-0627-z PMC5486798

[28] Scott DL Wolfe F Huizinga TW. Rheumatoid arthritis. Lancet. 2010;376:1094–108. 20870100 10.1016/S0140-6736(10)60826-4

[29] Bernstein EJ Barr RG Austin JHM. Rheumatoid arthritis-associated autoantibodies and subclinical interstitial lung disease: the multi-ethnic study of atherosclerosis. Thorax. 2016;71:1082–90. 27609750 10.1136/thoraxjnl-2016-208932 PMC5342945

[30] Shimizu T Nagafuchi Y Harada H. Decreased peripheral blood memory B cells are associated with the presence of interstitial lung disease in rheumatoid arthritis: a case-control study. Mod Rheumatol. 2021;31:127–32. 32023138 10.1080/14397595.2020.1719596

[31] Atkins SR Turesson C Myers JL. Morphologic and quantitative assessment of CD20+ B cell infiltrates in rheumatoid arthritis-associated nonspecific interstitial pneumonia and usual interstitial pneumonia. Arthritis Rheum. 2006;54:635–41. 16447242 10.1002/art.21758

[32] Lai NL Jia W Wang X. Risk factors and changes of peripheral NK and T cells in pulmonary interstitial fibrosis of patients with rheumatoid arthritis. Can Respir J. 2019;2019:7262065. 31885749 10.1155/2019/7262065 PMC6914899

[33] Shah Gupta R Koteci A Morgan A George PM Quint JK. Incidence and prevalence of interstitial lung diseases worldwide: a systematic literature review. BMJ Open Respir Res. 2023;10:e001291. 10.1136/bmjresp-2022-001291 PMC10277538 37308252

[34] Juge PA Lee JS Ebstein E. MUC5B promoter variant and rheumatoid arthritis with interstitial lung disease. N Engl J Med. 2018;379:2209–19. 30345907 10.1056/NEJMoa1801562 PMC6371965

[35] Cavagna L Monti S Grosso V. The multifaceted aspects of interstitial lung disease in rheumatoid arthritis. Biomed Res Int. 2013;2013:759760. 24205507 10.1155/2013/759760 PMC3800606

